# The histone-methyltransferase DOT1L cooperates with LSD1 to control cell division in blast-phase MPN

**DOI:** 10.1038/s41375-025-02719-y

**Published:** 2025-08-08

**Authors:** Karl Kapahnke, Thomas Plenge, Tabea Klaus, Manoj K. Gupta, Disha Anand, Tamer T. Önder, Birgit Perner, Tina M. Schnöder, Felicitas R. Thol, Frederik Damm, Florian H. Heidel, Florian Perner

**Affiliations:** 1 Hematology, Hemostasis, Oncology, and Stem Cell Transplantation, Hannover Medical School, Hannover, Germany; 2 University Medicine Greifswald, Greifswald, Germany; 3 School of Medicine, Koc University, Istanbul, Turkey; 4 Department of Hematology, Oncology and Tumor Immunology, Charité University Hospital Berlin, Berlin, Germany; 5 Leibniz Institute on Aging, Fritz-Lipmann-Institute, Jena, Germany; 6 Cellular Therapy Center, Hannover Medical School, Hannover, Germany

**Keywords:** Preclinical research, Myeloproliferative disease

## Abstract

Persistence of JAK2-mutated clones that may undergo clonal evolution and malignant transformation remains a challenge in myeloproliferative neoplasms (MPN), Novel therapeutic approaches to attenuate clonal evolution and progression to blast-phase are therefore urgently needed. LSD1 (KDM1A) inhibitors reduce symptoms and clonal burden in MPN, but whether these compounds may be effective in advanced disease stages remained so far elusive. Using a chromatin-focused CRISPR-Cas9 screen, we identified the histone methyltransferase DOT1L as a synthetic lethal target under pharmacologic LSD1 inhibition. DOT1L knockout impaired cellular fitness, reduced proliferation, and prolonged survival in xenografts. Furthermore, genetic inactivation of DOT1L increased LSD1 inhibitor sensitivity up to 100-fold resulting in cell cycle arrest and apoptosis induction in TP53 mutant blast-phase MPN. Mechanistically, we have identified a novel, non-canonical function of DOT1L which co-occupied LSD1-bound enhancers and contributed to the repression of transcriptional programs independent of its enzymatic activity. DOT1L loss cooperated with LSD1 inhibitors to activate tumor suppressive programs, while pharmacologic inhibition of DOT1Ls catalytic activity failed to elicit comparable effects. These findings indicate that leveraging DOT1L targeting via protein degradation or RNA interference, rather than conventional enzymatic inhibition, could enhance the therapeutic efficacy of LSD1 inhibitors in blast-phase MPN.

## Introduction

Activating mutations in Janus kinase 2 (JAK2)-, the thrombopoietin receptor- or the Calreticulin-gene cause constitutive activation of the JAK-STAT pathway and are oncogenic drivers of classic myeloproliferative neoplasms (MPN), like Polycythemia vera (PV), Essential thrombocythemia (ET) and Myelofibrosis (MF) [1, 2]. JAK inhibitors have demonstrated efficacy in managing inflammation and hyperproliferation in MPN [3] and are approved for the treatment of PV and MF [4]. However, they fail to eliminate JAK2-mutated clones, which can persist and potentially undergo clonal evolution, leading to disease progression [5]. For MPN high-risk patients [6], particularly those progressing to blast-phase MPN, effective control of the malignant clone remains a significant challenge as current treatment regimens, including allogeneic stem cell transplantation, have shown limited success [7, 8]. This issue is compounded by the advanced age of many patients limiting the applicability of intensive treatment options. Therefore, effective and well-tolerated targeted therapies for patients with high-risk MPN and especially those with accelerated and blast-phase MPN are urgently needed [6].

Small-molecule inhibitors targeting lysine-specific demethylase 1 (LSD1/KDM1A), such as Bomedemstat, have emerged as promising therapeutic candidates in MPN [9]. Clinical trials have demonstrated disease-modifying potential, including significant reductions in inflammatory symptoms, spleen size and clonal burden in a substantial proportion of patients [10–12]. However, the extent to which LSD1 inhibitors can maintain their therapeutic efficacy during progression to blast-phase MPN remains unclear. In early-phase clinical trials, LSD1 inhibitors showed only modest activity in Acute Myeloid Leukemia (AML) [13–15], indicating that effective combination therapy approaches will be required to achieve clinically meaningful responses in aggressive myeloid blood cancers.

In this project, we therefore aimed to evaluate epigenetic co-vulnerabilities of LSD1 in blast phase MPN with the goal to identify targetable dependencies that would increase the potency of LSD1 inhibitor treatment.

## Material and methods

### CRISPR-Cas9 screening

HEL cells were stably transduced with Lenti-Cas9-2 A-Blast (Addgene #73310) and single clones were selected and screened for rapid depletion of cells co-transduced with a single-guide RNA (sgRNA) targeting CDK9 as a common essential gene. The best performing clone was expanded, and 100 Mio cells were transduced with the EPIKOL [16] library at low multiplicity of infection (MOI). Puromycin selection (1 µg/ml) was performed starting 48 h after transduction and cells were constantly cultured in Puromycin and Blasticidin (10 µg/ml). After 3 d of selection cells were divided in 12 groups and treated with Bomedemstat (50 nM), GSK2879552 (50 nM) or DMSO as control (4 replicates / condition). After 14 d, cells were harvested, genomic DNA was isolated using the DNA Blood Mini Kit (Qiagen, Hilden, Germany) and CRISPR-screening analysis was performed as previously described [17].

### Generation of DOT1L-knockout lines

HEL cells stably expressing Cas9 were generated as described above and transduced with sgRNAs targeting DOT1L (or empty vector control) in the pUSE sgRNA vector system [17] containing Puromycin resistance and BFP. To select single clones, 100 cells were plated in 3 ml Methocult^TM^ H4230 (Stemcell Technologies, Vancouver, Canada) supplemented with 10% FBS, Puromycin (1 µg/ml) and Blasticidin (10 µg/ml) in a 6-well plate. After 10-12 days, single colonies were picked, expanded and screened for BFP expression and H3K79me2 in Western Blot.

### Xenograft model

NOD-Prkdc^scid^-IL2rg^Tm1^/Rj (NXG) mice were purchased from Janvier Labs (Le Genest-Saint-Isle, France). Male and female animals of 8-12 weeks of age were maintained in groups in single-ventilated IVC-cages in the animal research facility of the University Medicine Greifswald (Germany). Sample size was determined by experimental experience with this model system and no blinding of investigators or specific methods for randomization were used. No animals were excluded from the analysis. The experimental conditions and procedures were approved by the authorities in the state of Mecklenburg–Western Pomerania (Landesamt für Landwirtschaft, Lebensmittelsicherheit und Fischerei / LALLF) under the proposal No.: 7221.3-1-024/21. For xenograft experiments, 50,000 HEL cells were injected into non-conditioned NXG mice by tail vein injection. Animals were monitored daily by inspection and weekly by structured scoring and the time to disease development was determined.

### Primary patient samples

Bone marrow samples from MPN-BP patients were processed and stored in the leukemia biobank at Hannover Medical School. This study was approved by the institutional review board of Hannover Medical School (ethical votes 936/2011 and 3432-2016) and written informed consent was obtained according to the Declaration of Helsinki.

### Western Blot of histone extracts

3Mio cells were washed in ice-cold PBS + Sodium Butyrate (5 mM). Nuclear extraction was performed by resuspension in Triton Extraction Buffer (TEB: PBS + 0.5% TritonX 10, 2 mM PMSF, 0.02% NaN3) and incubation for 10 min. at 4 °C followed by centrifugation (650xg,10 min, 4 °C). Nuclei were washed with 500 µl TEB and resuspended in 75 µL HCl (0.2 N) and incubated at 4 °C overnight. Histone extracts were equally loaded on 15% acrylamide gels, electrophoresis and Western blotting were performed and blots were probed with monoclonal antibodies (Cell Signaling Technologies, Danvers, MA, USA) against H3K79me2 (clone: D15E8), total Histone H3 (clone: D1H2) or DOT1L (clone: D1W4Z).

### Cell growth assays

For cell competition assays [17] WT or DOT1L-ko single clones (BFP + ) were mixed with non-transduced Cas9-expressing competitor cells and the chimerism was determined by flow cytometry. The change in BFP-chimerism was then followed on days 3, 6, 10 and 15 and the relative change to baseline was calculated. Dose-response assays were performed using a colorimetric MTS assay (Promega, Madison, WI, USA) at 16 days of incubation with LSD1 inhibitors at 4.9 nM, 9.8 nM, 19.5 nM, 39.1 nM, 78.1 nM, 156.3 nM, 312.6 nM, 625 nM, 1250 nM or DMSO as control. To determine cell growth at fixed doses over a longer period, 150,000 cells were plated in 24 well plates and cell numbers were counted every 3-4 days before splitting. Based on these counts and the number of passaged cells a total cell count was calculated over the course of 30 days.

### Apoptosis

Apoptosis induction was measured using flow cytometry by staining cells with AnnexinV-AF647 (Biolegend, San Diego, CA, USA) and Sytox-blue dead cell stain (Thermo Fischer Scientific, Waltham, MA, USA) according to the manufacturer’s instructions.

### Cell cycle analysis

Cell cycle analysis was performed using the Click-iT™ Plus EdU Alexa Fluor™ 647 Flow-cytometry kit (Thermo Fischer Scientific, Waltham, MA, USA). EdU incorporation was performed for 2 h before cell harvest and subsequent sample preparation was conducted according to the manufacturer’s instructions.

### RNA-sequencing

Total RNA was isolated using the RNeasy Mini Kit (Qiagen). Messenger-RNA (mRNA) was purified using the NEBNext® Poly(A) mRNA Magnetic Isolation Module followed by RNAseq library preparation using the NEBNext® Ultra™ RNA Library Prep Kit for Illumina® (New England Biolabs, Ipswich, MA, USA) according to the manufacturer’s instruction. Sequencing was performed on the NovaSeqXPlus platform (Illumina, San Diego, CA, USA). Analyses were performed using an in-house Galaxy pipeline hosted at Hannover Medical School (MHH). A detailed description of the experimental procedures and bioinformatic analysis is provided in supplementary material and methods.

### ChIP-sequencing

Chromatin Immunoprecipitations (ChIP) and computational analyses were performed as described in detail in supplementary material and methods. For IPs we used antibodies against DOT1L (Cell Signaling Technologies, clone: D1W4Z), LSD1 (Abcam, clone: EPR6825), H3K4me1 (Abcam, clone: EPR16597) and H3K79me2 (Cell Signaling Technologies, clone: D15E8). Sequencing was performed on the NovaSeqXPlus platform (Illumina). A detailed description of the experimental procedures and bioinformatic analysis is provided in supplementary material and methods.

### Statistics

Kaplan-Meier curves, bar graphs and dot plots were plotted using GraphPad Prism (GraphPad Software, San Diego, CA, USA). Survival was analyzed using the log-rank test (Mantel-Cox test). Other statistical analyses were performed using a student t test or One-way ANOVA. A value of *p *< 0.05 was considered statistically significant.

## Results

### A CRISPR-Cas9 screen revealed synthetic lethal vulnerabilities under LSD1 inhibitor treatment

**Fig. 1:**
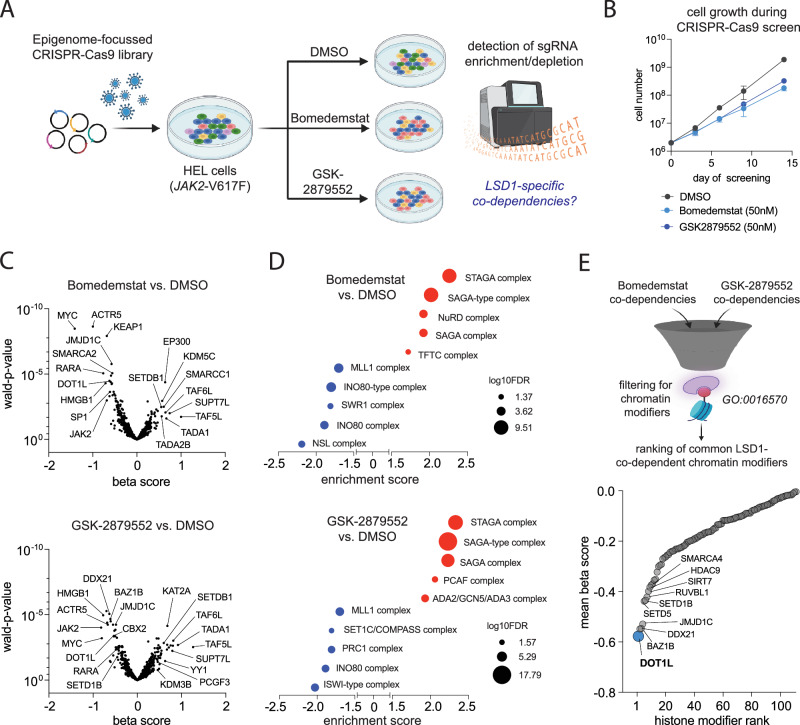
CRISPR–Cas9 screen under LSD1 inhibitor treatment. **A** Schematic depiction of the workflow to conduct an epigenome-focused CRISPR-Cas9 screen using the EPIKOL-library in HEL cells. **B** Growth curve of HEL cells treated with Bomedemstat (50 nM), GSK2879552 (50 nM) or DMSO for 14 days during the CRISPR-Cas9 screen (*n *= 4). **C** Volcano-plots showing differential dependencies of gene-targets from the EPIKOL-library under LSD1 inhibitor treatment. Beta-scores (x-axis) represent the directionality of gene-effects in the MaGECK-MLE analysis (LSD1 inhibitor vs. DMSO). Genes with negative beta-scores represent synthetic lethal candidates, positive beta scores identify putative resistance mediators. Wald-p-values are plotted on the y-axis. Plots show the results of the analysis of co-vulnerabilities under treatment with Bomedemstat (top) or GSK2879552 (bottom). **D** Enrichment analysis of Gene-ontology terms (GO cellular component) from positively (red) and negatively (blue) selected gene hits in the CRISPR-Cas9 screen under treatment with Bomedemstat (top) or GSK2879552 (bottom). Analysis was performed using String-db. Size of dots is proportional to the confidence in each term (log10FDR). **E** Identification of consensus synthetic lethal candidate hits. Schematic of filtering strategy (top) and ranking of consensus hits (bottom). Mean beta score = (beta_Bomedemstat_ + beta_GSK2879552_) / 2.

To identify epigenetic co-dependencies of LSD1 in blast-phase MPN, we conducted a chromatin-focused CRISPR-Cas9 screen in the “Human Erythroleukemia” (HEL) cell line. HEL cells harbor multiple copies of JAK2-V617F as the underlying MPN-driver mutation and a loss of p53, a genetic constellation that is typical and critical for progression and dismal prognosis of blast-phase MPN [7, 8, 18]. To avoid bias, we conducted the CRISPR-Cas9 screen using the two clinically available LSD1 inhibitors, Bomedemstat and GSK2879552 [11, 19]. HEL cells were transduced with the EPIKOL sgRNA-library [16] containing 7856 sgRNAs targeting 719 chromatin-related genes and cells were exposed to Bomedemstat, GSK2879552 or DMSO as control for 14 days (Fig. 1A). The screen was conducted at moderate drug concentrations of 50 nM, which impaired the proliferation rate while still allowing exponential growth without apoptosis induction, mirroring suboptimal treatment efficacy (Fig. 1B).

A substantial overlap of the most significant synthetic lethal interactors and resistance mediators could be observed between both LSD1 inhibitors used, indicating high on-target activity (Fig. 1C, Supplementary Table 1). Interestingly, JAK2 itself scored in both screens as a synthetic lethal gene, confirming a cell-intrinsic, genotype-selective vulnerability of JAK2-V617F mutant cells for LSD1 inhibitor treatment. Similarly, gene ontology analysis (GO cellular component) revealed an almost identical set of protein complexes whose knockout caused resistance or sensitized HEL cells to LSD1 inhibitor treatment (Fig. 1D, Supplementary Table 2). Interestingly, knockout of SAGA/STAGA complex members consistently caused a decrease in LSD1-inhibitor sensitivity. This fact likely indicates a critical role of SAGA/STAGA complexes in mediating LSD1 inhibitor driven responses and raise the question if genetic or non-genetic impairment of this chromatin modifiers complex could be linked to LSD1 inhibitor resistance.

Among the chromatin-related proteins that are synthetic lethal with LSD1 inhibition our screen identified INO80 chromatin remodeling, as well as MLL/COMPASS-like complexes in the context of both inhibitors (Fig. 1D). To identify targetable histone modifiers that become synthetic lethal vulnerabilities under LSD1 inhibitor treatment we first filtered our CRISPR-screening results for annotated chromatin modifiers (Gene ontology: histone modification; GO:0016570), calculated a mean CRISPR-beta-score for Bomedemstat and GSK2879552 and ranked the top100 synthetic lethal hits in an ascending order (Fig. 1E). This analysis revealed the histone methyltransferase “Disruptor Of Telomeric Silencing 1-Like” (DOT1L) as the strongest synthetic lethal consensus hit, indicating functional cooperation between LSD1 and DOT1L in blast-phase MPN.

### DOT1L mediates cell competition and oncogenic gene expression in blast-phase MPN

**Fig. 2:**
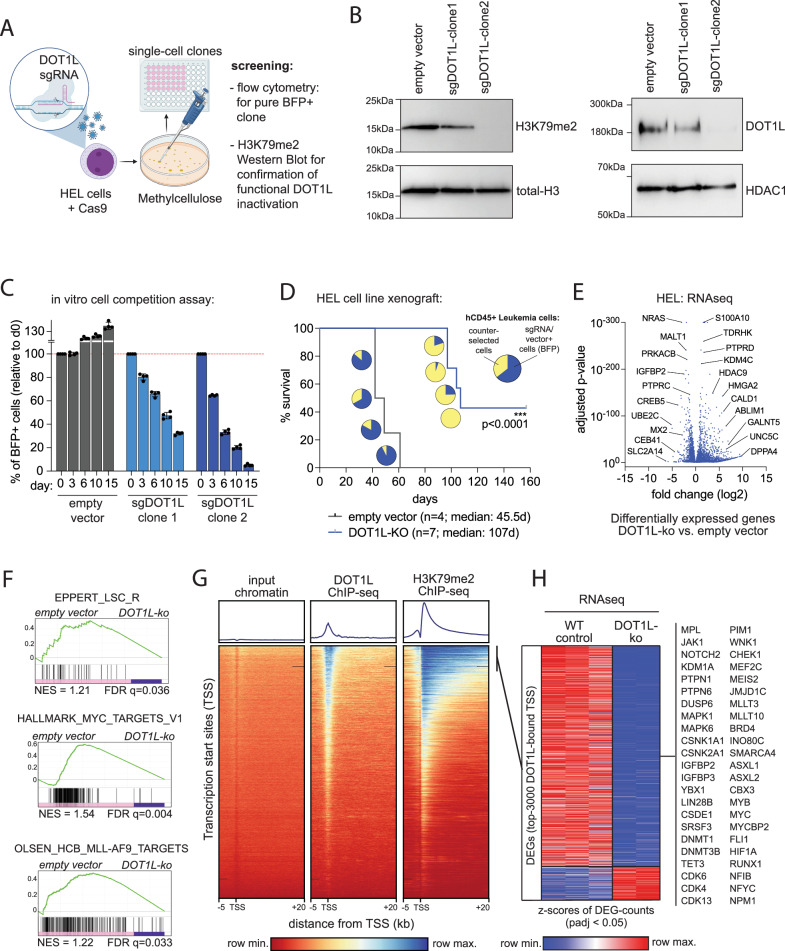
DOT1L mediates cell competition and gene expression. **A** Illustration of the experimental strategy to isolate DOT1L-ko clones. **B** Western blot showing global H3K79me2 abundance in histone extracts (right) or DOT1L protein levels in nuclear extracts (left) from DOT1L-ko HEL clones with partial or total loss of DOT1L activity (sgDOT1L-clone 1; sgDOT1L-clone 2). **C** Bar graphs showing the results of a fluorochrome-based cell competition assay. Relative abundance of empty vector control or DOT1L-sgRNA expressing HEL cells as compared to non-transduced competitor cells in a chimeric mixture are shown. **D** Kaplan–Meyer curves showing survival of NXG mice engrafted with 50,000 HEL cells harboring a complete DOT1L-ko (clone 2) or empty vector control. Pie charts show the fraction of sgRNA vector expressing cells in human CD45+ cells in the bone marrow of mice that developed disease, indicating counterselection of the BFP+ cells in the animals of the DOT1L-ko cohort. Statistics: log-rank test, ***p < 0.0001. **E** Volcano-plot showing differentially expressed genes (DEGs) from RNAseq in HEL cells after knockout of DOT1L (clone 2). **F** Geneset-enrichment analysis plots from RNAseq in HEL cells after knockout of DOT1L. **G** Tornado-plots visualizing the global occupancy of DOT1L and deposition of H3K79me2 across all transcription start sites (TSS) in ChIPseq of HEL cells. **H** Heatmap showing the fractions of UP- and DOWN-regulated DEGs after DOT1L-ko among those genes associated with the top 3000 DOT1L-bound TSS. Selected genes from the downregulated cluster are annotated.

DOT1L is the only known methyltransferase that catalyzes mono-, di- and tri-methylation of lysine 79 on histone H3 (H3K79me1/2/3) and has been identified as a critical component of oncogenic “Mixed Lineage Leukemia 1” (MLL1/KMT2A) complexes in KMT2A-rearranged and NPM1c mutant acute leukemia [20–23]. Potent and selective small molecule inhibitors of DOT1L have been developed and tested in preclinical and early-phase clinical trials and showed good tolerability but rather modest efficacy in patients with MLL-rearranged leukemia [24–26]. So far, DOTL had never been described to have a role in chronic- or blast-phase MPN. Therefore, we aimed to evaluate its function in MPN-BP (HEL) cells. First, we generated stable DOT1L-knockout lines (Fig. 2A). Two clones with a partial (sgRNA1, clone 1) or complete (sgRNA2, clone 2) loss of DOT1L activity, indicated by global loss of the H3K79me2 mark in Western blot of histone extracts, and loss of the DOT1L protein in nuclear extracts were identified (Fig. 2B). To determine the impact of DOT1L-deficiency on cell growth, we performed a cell competition assay in which complete and partial loss of DOT1L activity led to a reduction in competitive cell growth in vitro (Fig. 2C). To evaluate leukemogenicity, we injected 50,000 HEL cells harboring a DOT1L knockout (clone 2) or non-targeting vector control into non-conditioned NXG-mice and the time to disease development was determined. As expected, injection of DOT1L-WT HEL cells (empty vector) led to the development of aggressive and fully penetrant blast-phase MPN (*n *= 4, median survival: 45.5 days). In contrast, DOT1L knockout significantly prolonged survival (*n *= 7, median survival: 107 days) and reduced disease penetrance with 40% of mice surviving beyond 150 days after transplantation (Fig. 2D). RNA-sequencing (RNAseq) analysis of DOT1L-ko (clone 2) vs. WT (empty vector) cells revealed dramatic global changes in gene expression after DOT1L inactivation (Fig. 2E, Supplementary Table 3). In gene set enrichment analysis (GSEA) DOT1L-ko HEL cells had lost leukemia stem cell (LSC) features, MYC-targets and the expression of KMT2A target genes (Fig. 2F). In “Chromatin Immunoprecipitation followed by Sequencing” (ChIPseq) we found DOT1L bound to about 30% of all transcription start sites (TSS) across the genome and as expected, loci with high DOT1L TSS-loading showed a strong H3K79me2 signal spreading into gene bodies of the bound targets (Fig. 2G). Most genes with high DOT1L loading and a strong and spreading H3K79me2 signal were down-regulated upon DOT1L-knockout validating its known function as a transcriptional co-activator at promoters (Fig. 2H). Of note, among these genes were critical regulators of cell cycle progression (MYC, MYB, MYCBP1, CHEK1), transcription factors (RUNX1, NFYC, NFIB, HIF1A, MEIS2), epigenetic modifiers (DNMT1, DNMT3B, TET3, BRD4, SMARCA4, INO80C) as well as established JAK-signaling targets and mediators of MPN-progression and persistence under JAK inhibitor treatment (YBX1, DUSP6, PIM1, ASXL1, BRD4) [27–31].

We further aimed to validate the critical function of DOT1L for gene regulation in primary human blast-phase MPN cells. We therefore validated a set of shRNAs for RNA-interference-based knockdown of DOT1L and selected two constructs (shRNA5 and shRNA6) for further experiments (Supplementary Fig. 1A). Cryo-preserved blast-phase MPN cells were thawed and transduced with lentiviral particles for the expression of DOT1L shRNA 5/6 or scambled shRNA as control and cultured in methylcellulose supplemented with puromycin for selection of successfully infected cells for 7 days (Supplementary Fig. 1B). Afterwards cells were harvested and subjected to Western blotting for the confirmation of DOT1L knockdown efficiency (Supplementary Fig. 1C) and RNA-sequencing. Of note, genetic inactivation of DOT1L in blast-phase MPN led to a change in cell identity features, including loss of erythroid lineage genes and HOX-genes as well as an upregulation of programs related to antigen presentation and immune activation (Supplementary Fig. 1D,E).

In summary, these experiments revealed a critical function of DOT1L for cell fitness and maintenance of oncogenic gene expression in blast-phase MPN.

### Loss of DOT1L sensitizes blast-phase MPN to LSD1 inhibition

**Fig. 3:**
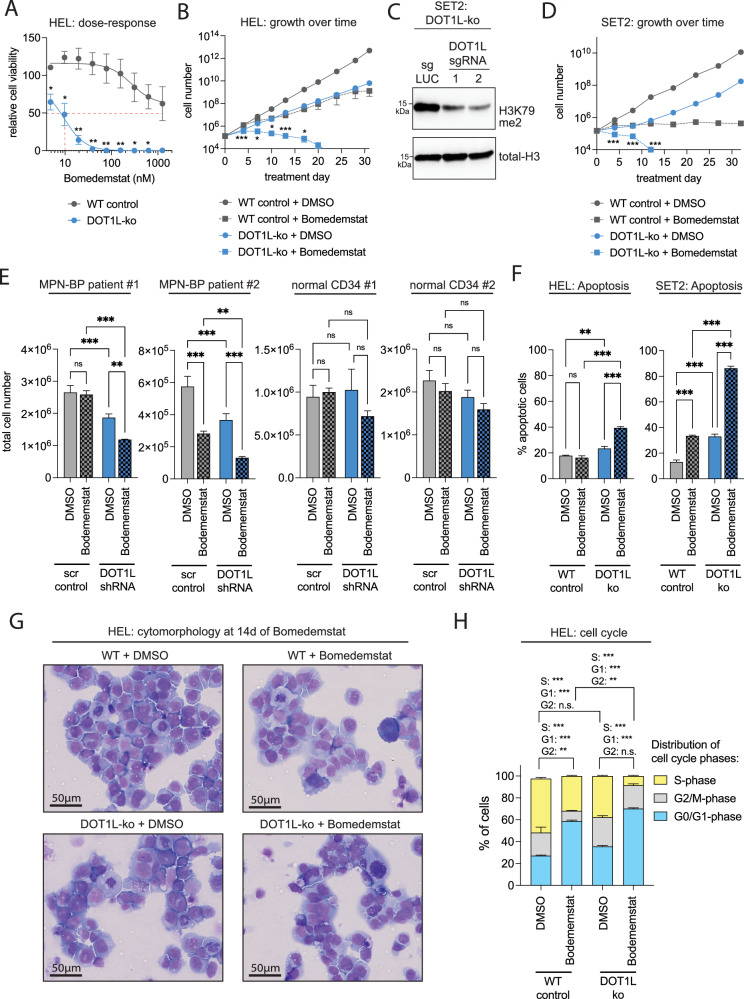
Loss of DOT1L sensitizes blast-phase MPN to LSD1 inhibition. **A** Dose-response curve showing the sensitivity of HEL cells to Bomedemstat treatment at 6 days of incubation determined by MTS assay; *n *= 3 independent experiments, unpaired t-test; *<0.05, **<0.01, ***<0.001. **B** Cell growth of HEL cells (empty vector vs. DOT1L-ko, clone 2) in a growth-over-time assay under exposure to Bomedemstat (150 nM); unpaired t-test; comparison of DOT1L-ko+Bomedemstat with WT+Bomedemstat; *<0.05, **<0.01, ***<0.001. **C** Western blot showing global H3K79me2 abundance in histone extracts from SET2 cells after CRISPR-Cas9-mediated inactivation of DOT1L with two different sgRNAs. **D** Cell growth of SET2 cells (Luciferase (LUC) sgRNA negative control vs. DOT1L-sgRNA2) in a growth-over-time assay under exposure to Bomedemstat (100 nM); unpaired t-test; comparison of DOT1L-ko+Bomedemstat with WT+Bomedemstat; ***<0.001. **E** Bar graphs showing the total number of primary cells from two different MPN-BP patients or G-CSF mobilized normal cells after shRNA-mediated knockdown of DOT1L (shRNA5/6) or scrambled (scr) control and treatment with 100 nM Bomedemstat or DMSO for 7 days in methylcellulose; one-way ANOVA; **<0.01, ***<0.001. **F** Bar graphs showing the fraction of apoptotic cells (HEL empty vector vs. DOT1L-ko, clone 2) using an AnnexinV / Sytox blue assay under exposure to Bomedemstat (150 nM) or DMSO at 14 d; *n *= 3 independent experiments, One-way ANOVA; **<0.01, ***<0.001. **G** Representative cytospins (May-Grünwald/Giemsa staining) from HEL cells (empty vector or DOT1L-ko, clone 2) under exposure to Bomedemstat (150 nM) or DMSO at 14 d. Imaging of whole slides was performed on a Carl Zeiss AxioScan. *n *= 3 independent experiments. Scale bar indicates 50 µM. **H** Stacked bar graphs showing the fraction of cells in G0/G1, G2/M and S-phase in HEL cells (empty vector vs. DOT1L-ko, clone 2) determined by Click-it EdU cell cycle assay under exposure to Bomedemstat (150 nM) or DMSO at 14 d; *n *= 3 independent experiments, One-way ANOVA; **<0.01, ***<0.001.

To validate the results from our CRISPR-Cas9 screen and confirm a role of DOT1L in mediating LSD1 inhibitor sensitivity, we next performed dose-response assays of DOT1L-ko and -WT HEL cells against Bomedemstat and GSK2879552. Knockout of DOT1L significantly sensitized cells to LSD1 inhibitor treatment reducing the concentration at which 50% growth inhibition is achieved from >1 µM to 10 nM and 20 nM, respectively (Fig. 3A, Supplementary Fig. 2A). Either DOT1L-knockout or LSD1 inhibitor treatment at moderate doses (Bomedemstat: 150 nM, GSK2879552: 100 nM) reduced the proliferation rate of HEL cells but allowed for long-term exponential growth. In contrast, LSD1 inhibitor treatment of DOT1L-knockout cells resulted in a complete blockade of cell growth (Fig. 3B, Supplementary Fig. 2B). Interestingly, also the incomplete genetic inactivation of DOT1L (sgDOT1L-clone1, Fig. 2B) had a significant impact on the fitness of cells treated with LSD1 inhibitors indicating a strong dependency of MPN-BP cells on high DOT1L expression levels (Supplementary Fig. 2C-F).

Furthermore, we performed CRISPR-Cas9-based genetic inactivation of DOT1L in SET2 cells, another JAK2-V617F/TP53 double mutant blast-phase MPN model (Fig. 3C) and were able to confirm that DOT1L-knockout cells showed a significant increase in LSD1-inhibitor sensitivity (Fig. 3D). Next, we employed our methylcellulose-based culture system (Supplementary Fig. 1B) to evaluate cell growth of two different primary MPN-BP patient samples and normal CD34+ cells from G-CSF mobilized peripheral blood mononuclear cells after RNAi-mediated knockdown of DOT1L and LSD1 inhibitor treatment. Of note, a significant cooperative growth inhibition could only be observed in MPN-BP samples, not in normal CD34+ cells demonstrating a disease specific therapeutic index despite comparable knockdown efficiency (Fig. 3E, Supplementary Fig. 1C and 2G).

The growth arrest observed in MPN-BP cell lines was accompanied by a significant increase in apoptosis induction in DOT1L-ko cells treated with Bomedemstat (Fig. 3F). In KMT2A-rearranged AML, LSD1-inhibitior activity had been linked to the induction of differentiation [32, 33]. To evaluate whether a similar mechanism is operative in blast-phase MPN we conducted cytomorphological analysis. DOT1L-knockout appeared to increase the frequency of atypical mitosis figures and polynucleated cells while Bomedemstat treatment discretely reduced the nucleus-plasma relation, but overall, the cellular morphology was largely comparable between the conditions (Fig. 3G). Most importantly, DOT1L-ko cells treated with Bomedemstat showed no indication of a greater degree of differentiation induction compared to WT cells exposed to the inhibitor (Fig. 3G, bottom right).

To determine the cause of the reduced proliferative output, we conducted a flow-cytometry based cell cycle assay using EdU (5-ethynyl-2′-deoxyuridine) incorporation and DNA content staining [34]. LSD1 inhibitor treatment led to a partial delay in cell cycle progression with an accumulation of cells in G0/G1 phase and a delayed but maintained S-phase transition (Fig. 3H). In contrast, in DOT1L-knockout HEL cells Bomedemstat led to a near-complete blockade of S-phase entry, explaining the severe growth arrest observed in cell growth assays (Fig. 3H). Therefore, we conclude that the attenuation of cell growth in DOT1L-ko cells treated with Bomedemstat is primarily caused by cell cycle arrest and apoptotic cell death rather than by induction differentiation.

### DOT1L-deficiency augments gene expression changes under LSD1 inhibition

**Fig. 4:**
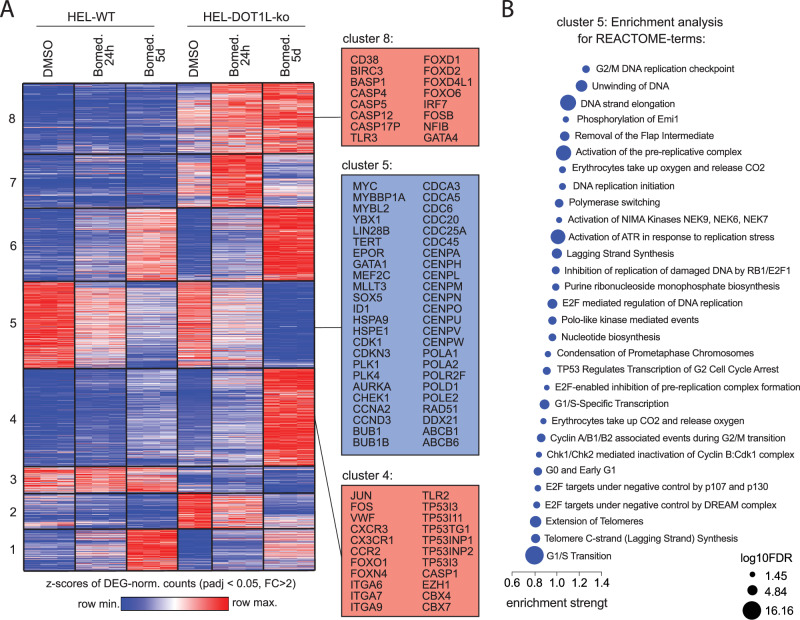
DOT1L-deficiency augments gene expression changes under LSD1 inhibition. **A** Heatmap showing differentially expressed genes (adjusted p-value < 0.05, fold-change >2) in RNAseq from HEL cell lines (empty vector or DOT1L-ko, clone 2) under exposure to Bomedemstat (150 nM) or DMSO at 24 h or 5 d. Based on the expression pattern, genes were assigned to 8 unique clusters (Kmeans clustering). Selected genes from clusters 4, 5 and 8 are annotated. **B** Enrichment analysis of REACTOME among genes strongly DOWN-regulated by Bomedemstat in DOT1L-ko cells (cluster 5). The size of dots is proportional to the confidence in each term (log10FDR).

DOT1L-ko or -WT cells were treated with DMSO or Bomedemstat (150 nM) for 24 h and 5 d and transcriptomes were analyzed by RNA sequencing. Differentially expressed genes (DEGs) were determined between all conditions using the DESeq2 algorithm (Supplementary Table 7), z-scores were calculated, a heatmap showing all DEGs (adjusted *p* value < 0.05, fold-change >2) in any of the comparisons was plotted and genes were assigned to 8 clusters (Kmeans clustering, Supplementary Table 8). Of note, in most clusters LSD1 inhibitor driven gene expression changes were augmented in DOT1L-knockout cells (Fig. 4A). In clusters 4 and 6, genes that were only discretely induced by Bomedemstat in WT cells became strongly upregulated when DOT1L-ko cells were treated with the drug. Moreover, in clusters 7 and 8 a subset of genes that were not induced in WT cells were upregulated in DOT1L-ko cells upon LSD1 inhibitor treatment. Among the genes with augmented induction in DOT1L-ko cells were members of protein complexes with known tumor suppressor functions (AP1, p53, forkhead transcription factors) as well as apoptosis mediators (CASP1, CASP4, CASP5, CASP10, TLR2, TLR3) (Fig. 4A).

Conversely, in cluster 5, genes that showed a mild decline in expression under LSD1-inhibition in WT cells are rapidly and potently silenced by Bomedemstat in DOT1L-ko cells. Interestingly, the cooperatively downregulated genes in cluster 5 were strongly enriched for cell cycle and cell division associated terms (Fig. 4B) providing a molecular explanation for the cell cycle arrest observed in functional assays. On a global transcriptome scale, the DOT1L-knockout alone only showed a relatively narrow spectrum of uniquely differentially regulated genes but strongly augments transcriptomic changes inflicted by the LSD1 inhibitor Bomedemstat.

### LSD1 and DOT1L co-occupy enhancers and cooperate to repress target genes

**Fig. 5:**
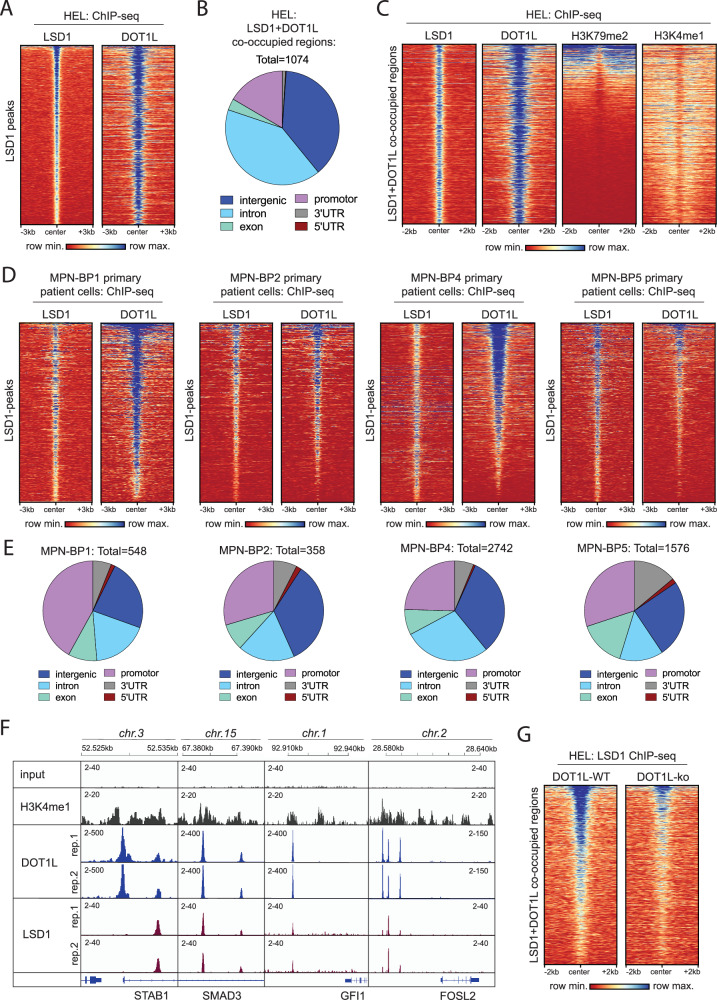
LSD1 and DOT1L co-occupy enhancers in MPN-BP cells. **A** Tornado-plots visualizing the occupancy of DOT1L at LSD1 binding sites in HEL cells. The map was created based on regions with LSD1-ChIPseq peaks (MACS2) and sorted by LSD1 signal intensity. DOT1L-ChIPseq signals were mapped to these regions showing a large degree of co-occupancy. **B** Pie-chart showing the assignment of LSD1 and DOT1L co-occupied sites to different regions in the genome (ChIPseeker output). **C** Tornado-plots visualizing the deposition of H3K79me2 and H3K4me1 at LSD1 and DOT1L co-occupied regions. Map was sorted based on H3K79me2 signal intensities. **D** Tornado-plots visualizing the occupancy of DOT1L at LSD1 binding sites in primary MPN-BP patient samples. The map was created based on regions with LSD1-ChIPseq peaks (MACS2) and sorted by LSD1 signal intensity. Regions with signal enrichment in the corresponding input samples were deemed artefacts and removed. DOT1L-ChIPseq signals were mapped to these regions showing a large degree of co-occupancy. **E** Pie-chart showing the assignment of LSD1 and DOT1L co-occupied sites in MPN-BP samples to different regions in the genome (ChIPseeker output). **F** Genome-browser (IGV / Integrative Genome Viewer) tracks showing DOT1L, LSD1 and H3K4me1 co-occupancy at selected regulatory regions in HEL cells. **G** Tornado-plots visualizing LSD1-signal intensity at LSD1 + DOT1L co-occupied genomic sites in DOT1L-WT and DOT1L-ko HEL cells.

We performed ChIPseq experiments in HEL cells to identify and compare genome-wide binding sites of LSD1 and DOT1L on chromatin, respectively. Aside from DOT1Ls expected association with transcriptionally active promoters (Fig. 2G) we observed occupancy of DOT1L at almost all regions at which LSD1 was bound (Fig. 5A). As anticipated for LSD1 binding sites, these regions did not primarily map to promoters but to introns or the intergenic space (Fig. 5B). The expectation that these regions are largely LSD1-bound enhancers was substantiated by the observation that the majority of LSD1 and DOT1L co-occupied sites were marked by histone 3 lysine 4 monomethylation (H3K4me1) (Fig. 5C). Interestingly, only a minority of the co-occupied genomic regions were marked by H3K79me2 indicating that the enzymatic activity of DOT1L may not be relevant at those enhancers.

In fact, only about a third of the DOT1L binding sites across the genome in HEL as well as SET2 cells were associated with H3K79me2 rather than with H3K4me1 (Supplementary Fig. 3A,B) demonstrating that enhancer binding of DOT1L represents a relevant proportion of DOT1L occupied regions. Interestingly, in *KMT2A*::*MLLT3* rearranged MOLM13 cells the same analysis revealed predominant binding of DOT1L to promoter regions and no relevant association with putative enhancers indicating a function in MPN cells that is distinct from KMT2A-rearranged leukemia (Supplementary Fig. 3C,D). In primary cells from MPN-BP patients the co-occupancy of LSD1 and DOT1L and the mapping of these regions outside of gene promoters could be confirmed, substantiating the observations made in the HEL cell line (Fig. 5D, E). DOT1L and LSD1 co-bound regions included several critical transcription factors and tumor suppressors (Fig. 5F) and knockout of DOT1L in turn led to reduction of LSD1 binding at these sites (Fig. 5G). Co-immunoprecipitations failed to confirm a direct physical interaction between DOT1L and LSD1, suggesting that they likely do not act within the same protein complex but rather cooperate at a subset of genomic loci without physically interacting with each other (Supplementary Fig. 4).

**Fig. 6:**
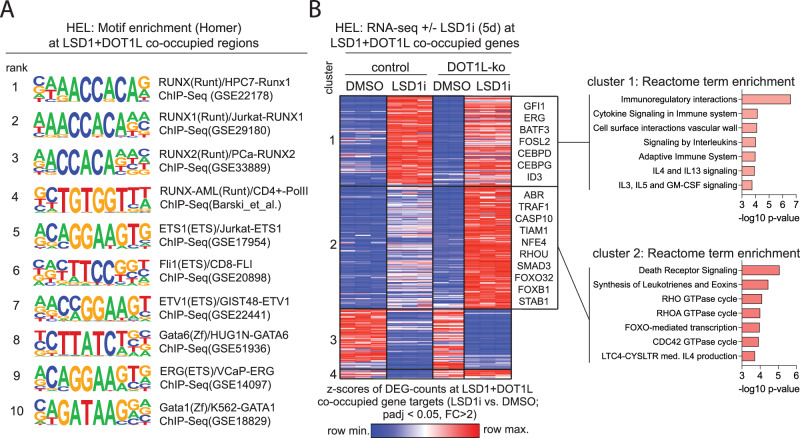
LSD1 and DOT1L cooperate to repress non-canonical target genes. **A** Top-10 transcription factor motifs enriched at LSD1 and DOT1L co-occupied regions in HEL cells. Output of the “Homer” motif enrichment analysis software. **B** Left: Heatmap showing DEGs (adjusted p-value < 0.05, fold-change >2) in RNAseq from HEL cell lines (empty vector or DOT1L-ko, clone 2) under exposure to Bomedemstat (150 nM) or DMSO at 5 d. DEGs were filtered for genes associated with LSD1 and DOT1L co-occupied regions (ChIPseeker). Genes were assigned to 4 clusters (Kmeans clustering). Selected genes from clusters 1 and 2 are annotated. Right: Enrichment analysis results of REACTOME terms in cluster 1 (top) and cluster 2 (bottom) are shown as bar graphs.

Enrichment analysis of LSD1 and DOT1L co-occupied regions for known transcription factor binding motifs revealed strong enrichment of RUNX, ETS and GATA motifs (Fig. 6A, Supplementary Fig. 5), most of which have previously been described to be associated with a LSD1/GFI1 repressor complex [32, 35–41]. To determine the function of DOT1L at these LSD1-bound enhancers, we filtered our RNAseq dataset for genes that (1) are in proximity to the co-occupied regions and (2) are differentially expressed upon LSD1 inhibitor treatment in either DOT1L-ko or -WT cells. Consistent with LSD1s known function as a transcriptional repressor, the majority of DEGs related to LSD1 binding sites were upregulated upon Bomedemstat treatment (Fig. 6B, clusters 1 + 2). A fraction of those genes was induced to a similar extent in DOT1L-ko and -WT cells, including several critical transcription factors relevant for myeloid maturation (Fig. 6B, cluster 1). Consequently, cluster 1 genes were enriched for functional terms associated with myeloid effector functions. Another subset of genes associated with LSD1 and DOT1L co-occupied regions was only discretely induced by LSD1-inhibition in WT cells but strongly upregulated in the DOT1L-ko background (Fig. 6B, cluster 2). In this subset of genes, a significant enrichment of cell death, cell cycle and cell polarization associated terms was observed. Therefore, loss of DOT1L from a subset of LSD1-bound enhancers seems to be required for potent induction of a network of tumor suppressive genes under Bomedemstat treatment. Particularly FOXO transcription factors and polarity mediators are interesting putative tumor suppressive effectors [42–49].

In summary, we have discovered a non-canonical function of DOT1L in which it co-occupies LSD1-bound enhancers contributing to transcriptional repression and protecting a subset of genes from transcriptional activation upon LSD1 inhibitor treatment. In DOT1L-ko cells, LSD1-inhibition unleashes the expression of a network of target genes to promote cell cycle arrest and apoptosis.

### Enzymatic inhibition of DOT1L fails to induce growth arrest of Bomedemstat-treated cells

**Fig. 7:**
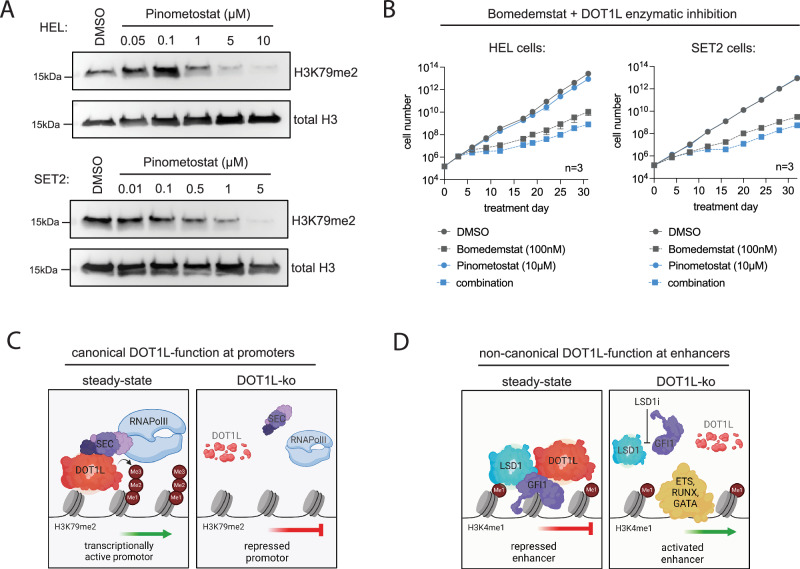
DOT1Ls cooperation with LSD1 is independent of its catalytic activity. **A** Representative Western blot showing global H3K79me2 abundance in histone extracts from HEL and SET2 cells treated with different concentration of the DOT1L inhibitor Pinometostat or DMSO as control. **B** Curves showing the cell growth of HEL or SET2 cells in a growth-over-time assay under exposure to single- or combination treatments with Bomedemstat (100 nM) and Pinometostat (10 µM). **C** Simplified model of DOT1Ls known canonical function at promoters of transcriptionally active genes. **D** Proposed working model of a novel non-canonical function of DOT1L at LSD1-bound enhancer regions.

The observation that LSD1 and DOT1L co-occupied regions in the genome largely lack H3K79-methylation, the histone mark deposited by DOT1L, was unexpected and interesting. To test if DOT1Ls enzymatic activity is relevant for its function in blast-phase MPN and whether co-treatment with DOT1L- and LSD1 inhibitors could be a viable therapeutic approach, we evaluated this combination in vitro. First, we determined the concentration of the DOT1L inhibitor Pinometostat (EPZ-5676) required to globally ablate H3K79me2 by Western blot analysis of histone extracts in HEL and SET2 cells (Fig. 7A). Surprisingly, the concentrations required for total or subtotal inhibition of DOT1L activity were relatively high, likely caused by particularly high expression levels of P-glycoprotein (MDR1/ABCB1; depmap.org), a known efflux transporter for Pinometostat [50]. We, therefore, used 10 µM of Pinometostat to conduct the subsequent functional experiments. In a growth-over-time assay over 30 d of culture, DOT1L inhibitor treatment failed to induce a growth arrest when combined with Bomedemstat (Fig. 7B). Furthermore, in contrast to the observations made after knockout of DOT1L (Figs. 2 and 3B, D) treatment with the Pinometostat alone did not inflict a relevant effect on cell growth. We, therefore, speculate that its enzymatic activity and the deposition of the H3K79me2 mark is dispensable for the function of DOT1L in blast-phase MPN.

Based on our observations we propose a model in which DOT1L has opposing functions at promoter and enhancer regions. At promoters, DOT1L is known to act as a positive regulator of transcription, deposit the H3K79-methylation mark and interact with the superelongation complex (SEC) to stimulate productive transcriptional elongation (Fig. 7C) [51–53]. In contrast, at enhancers DOT1L appears to have a so far unknown, non-canonical function: It occupies regions at which transcription factor activity is repressed by LSD1 complexes and loss of DOT1L, but not inhibition of its enzymatic activity, is required for the potent induction of transcriptional programs driving cell cycle arrest and apoptosis upon LSD1 inhibitor treatment (Fig. 7D).

## Discussion

The question for which functional properties of DOT1L its catalytic activity and the deposition of H3K79-methylation is relevant remains highly controversial. In KMT2A-rearranged leukemia the enzymatic activity of DOT1L appears to be relevant and inhibition shows therapeutic efficacy in preclinical model systems and modest activity in a phase-1 clinical trial [24–26, 54]. Nevertheless, the exact mechanistic underpinnings remain largely unknown. The fact that still no typical “reader” proteins for H3K79me have been identified further complicates the identification of bona-fide downstream pathways. A recent report indicates that the chromatin adapter protein Menin might serve as a reader for H3K79me2, potentially explaining the importance of this mark for leukemias driven by Menin-KMT2A complexes [55]. In contrast, studies using an enzyme-dead mutant of DOT1L in embryonic stem cells indicate that enzyme independent functions rather than deposition of H3K79-methylation are critical for its role in cell-fate determination [56]. In mice, both Dot1l-ko and the expression of an enzyme-dead mutant of Dot1l are embryonically lethal but defects in primitive hematopoiesis were largely rescued by methylation-incompetent Dot1l, indicating that non-enzymatic functions might be particularly relevant for hematopoietic cells [57].

Most studies investigating the role of DOT1L in transcriptional regulation have focused on its canonical function at promoters. So far only few reports have shown DOT1L binding to enhancers. In KMT2A::AFF1 rearranged leukemia, DOT1L-binding and consequent H3K79me was reported at a subset of enhancers [58]. In contrast to our findings, DOT1L acted as an activator of those enhancers in an enzyme-dependent manner and DOT1L inhibitor treatment led to repression of enhancer activity. To the best of our knowledge, the only indication of DOT1L acting as a repressor of enhancer function so far was reported from a study in C. elegans [59], yet the underlying mechanisms remained largely unresolved.

A functional interaction between the histone demethylase LSD1 and DOT1L has also not been established so far. Although DOT1L did appear as a synthetic lethal combination partner under LSD1-inhibition in a different CRISPR screen in AML [60], the cooperation between the two molecules had so far not been characterized in detail. Nevertheless, this line of evidence suggests that the interaction between LSD1 and DOT1L is likely not specific for JAK2-V617F mutant blast-phase MPN.

Although LSD1 inhibitors show promising disease modifying activity in clinical trials in chronic-phase MPN [10–12] the successful use of this class of drug in blast-phase will require potent combination therapy approaches. A particular challenge for the treatment of this subset of patients is the fact, that a large proportion of them carry TP53 loss-of-function mutations or deletions on both alleles [18]. These patients typically have a devastating prognosis under conventional chemotherapy and even show adverse outcomes after allogenic stem cell transplantation [7, 8]. Loss of TP53 has also been shown to impair apoptosis induction and therapeutic outcomes in AML models treated with LSD1 inhibitors [61]. Therefore, the fact that DOT1L-deletion rendered a highly aggressive TP53 mutant blast-phase MPN model sensitive to LSD1 inhibition causing growth arrest and even induction of apoptosis is remarkable and demands further preclinical exploration. The observation that enzymatic inhibition of DOT1L does not seem to elicit relevant therapeutic efficacy complicates this attempt. To overcome this limitation, RNAi-based targeting approaches or targeted protein degradation may be used to facilitate preclinical studies and eventually the translation to clinical trials. Of note, small molecule DOT1L-degrader molecules (PROTACs) are currently under development (Patent: WO2020/006157) and may be available for mechanistic and preclinical studies in the future.

## Supplementary information

Supplementary material and methods

Supplementary Tables

## Data Availability

All sequencing data are available via the Gene Expression Omnibus (GEO) portal under the accession numbers GSE300505 (ChIPseq) and GSE300512 (RNAseq). Other relevant processed datasets are available as supplementary tables (Supplementary Tables 1-19).
