# Supplementary material for: The histone-methyltransferase DOT1L cooperates with LSD1 to control cell division in blast-phase MPN
Source: Leukemia. 2025 Aug 8;39(10):2406–18. doi: 10.1038/s41375-025-02719-y (PMC12463660; doi:10.1038/s41375-025-02719-y)
Supplement: Supplementary file 1 — Supplementary material and methods [file 41375_2025_2719_MOESM1_ESM.pdf]

Supplementary Material & Methods:

The histone-methyltransferase DOT1L cooperates with LSD1 to control cell division in blast-phase MPN

Karl Kapahnke, Thomas Plenge, Tabea Klaus, Manoj K. Gupta, Disha Anand, Tamer T. Önder, Birgit Perner, Tina M. Schnöder, Felicitas Thol, Frederik Damm, Florian H. Heidel, Florian Perner

Chromatin-Immunoprecipitation and sequencing:

Cells were slow-spun to remove dead cells and washed once with PBS. Between 1 and 10 × 10^6 cells/mL were resuspended in PBS and subjected to protein–protein crosslinking by adding 50 µL/mL of 40 mM disuccinimidyl glutarate (DSG) for 30 minutes on a rotator at room temperature. Cells were then washed three times with PBS to completely remove DSG and resuspended in PBS at a concentration of 0.5–5 × 10^6 cells/mL.

For protein–DNA crosslinking, a 2% formaldehyde solution was added 1:1 to the cell suspension and rotated at room temperature for 10 minutes. The reaction was immediately quenched by adding 200 µL of 1.25 M glycine and 100 µL of 1 M Tris-HCl per mL of suspension. Cells were pelleted by centrifugation (1400 rpm, 5 min) and washed twice with cold PBS.

Cells were lysed in 1 mL of Cytoplasmic Lysis Buffer (20 mM Tris-HCl pH 7.5, 300 mM NaCl, 2 mM EDTA, 0.5% NP-40, 1% Triton X-100; freshly supplemented with 1 mM PMSF and cOmplete™ Protease Inhibitor Cocktail) and incubated on ice for 30 minutes. Nuclei were extracted by applying 10–15 strokes with a pre-chilled Dounce homogenizer, transferred into a fresh tube, and centrifuged at 2600 × g at 4 °C for 10 minutes.

The nuclear pellet was resuspended in Sonication Buffer (10 mM Tris-HCl pH 8.1, 100 mM NaCl, 1 mM EDTA, 1% Triton X-100, 0.5% N-lauroylsarcosine, 0.1% SDS; freshly supplemented with 1 mM PMSF and cOmplete™ Protease Inhibitor Cocktail). Chromatin was either stored at −80 °C or immediately sonicated to an average fragment size of 200–600 bp using a Covaris ME220 Focused-ultrasonicator (duration: 2100s, peak power: 75 W, 2100 s, duty factor: 15 %, cycles/burst: 1000). After sonication, lysates were centrifuged at maximum speed (≥13,000 × g) for 10 minutes at 4 °C, and the supernatant containing the fragmented chromatin was transferred to a

fresh tube. Sheared chromatin was either stored at −80 °C or directly used for immunoprecipitation.
For input control, 10 µL of chromatin were used and de-crosslinked in 150 µL Elution Buffer (50 mM Tris-HCl pH 8.0, 10 mM EDTA, 1% SDS) at 65 °C for 4 hours or overnight.
For immunoprecipitation, 5 µg of antibody per IP were used. Protein A Dynabeads (4.2 µL per 1 µg antibody) were washed twice in Sonication Buffer using a magnetic stand (DynaMag-2). Beads were then resuspended in 500 µL Sonication Buffer supplemented with 10 µL of 50 mg/mL BSA. Antibodies and beads were incubated for 1 hour at 4 °C on a rotator, then washed once with Sonication Buffer. The antibody–bead complex was resuspended in 50 µL of Sonication Buffer per IP and added to chromatin equivalent to 10 million cells (for DOT1L and LSD1) or 2 million cells (for histone marks). Samples were incubated overnight at 4 °C on a rotator.
Beads were then resuspended in Wash Buffer (50 mM HEPES pH 7.6, 500 mM LiCl, 1 mM EDTA, 1% NP-40, 0.7% sodium deoxycholate; freshly supplemented with 1 mM PMSF) and washed six times with 800 µL Wash Buffer by gentle inversion for 2 minutes per wash, followed by a final wash with 1 mL of 1x TE buffer. Chromatin was eluted in 150 µL Elution Buffer, incubated on a shaking heat block at 65 °C for 30 minutes, separated from beads using a magnet, and de-crosslinked for 4h or overnight at 65 °C. ChIP and input DNA were purified using 0.9x AmpureXP beads, washed twice with 80% ethanol, and eluted in 15–35 µL 0.1x TE buffer.
Library preparation for Illumina sequencing was performed using the NEBNext Ultra II RNA Library Prep Kit (NEB #E7770), following the manufacturer's instructions. Libraries were purified with 0.9× AmpureXP beads and PCR-enriched using the NEBNext Index/Universal Primer Mix (NEB #E6611A). ChIP and input DNA were quantified before and after amplification using Qubit (dsDNA HS). Ten to twelve amplification cycles were performed per sample, aiming for an increase in DNA concentration of ≥3 ng/µL per sample. Library size distribution was assessed using an Agilent TapeStation. Paired-end sequencing was carried out on an Illumina NovaSeq X-Plus platform (Genewiz/Azenta).

Computational data analysis:

ChIPseq:

Raw FASTQ files were uploaded to the Galaxy platform for analysis. Quality control was performed using FastQC (0.74) and adapter trimming (last 30 bases) was done with Fastp (0.23.4). Reads were aligned to the human reference genome (GRCh38/hg38) using Bowtie2 (2.5.3). Read counts were obtained using bedtools multicov (2.30.0), and coverage tracks were generated using bamCoverage (3.5.4) for visualization in IGV (2.17.4).

Duplicates were removed using macs2 filterdup (2.2.9.1) and peaks were called with macs2 callpeak (2.2.9.1) using the corresponding input samples as controls. Signal intensities ±3 kb around transcription start sites were computed with ComputeMatrix (3.5.4) and visualized using plotHeatmap (3.5.4). Peaks were annotated using ChIPseeker (1.28.3)

RNAseq:

Raw FASTQ files were processed on the Galaxy platform. Adapter trimming and quality filtering were performed using Fastp (0.23.4). Trimmed paired-end reads were aligned to the human reference genome GRCh38/hg38 using RNA STAR (2.7.11a). Mapped reads were quantified using featureCounts (2.0.3) against the corresponding GTF annotation file. Differential gene expression analysis was carried out using DESeq2 (2.11.40.8), and genes with an adjusted p-value < 0.05 and a fold change >2 were considered significantly differentially expressed.

Single-guide RNAs used for CRISPR-Cas9-mediated knockout of DOT1L:

DOT1L-sgRNA1:	GGGAGCGAATCGCCAACACG
DOT1L-sgRNA2:	GTCCACAAACAGGTCGTCGT

Misson® shRNAs (Sigma/Merck) for knockdown of DOT1L:

DOT1L-shRNA5:	TRCN0000020209
DOT1L-shRNA6:	TRCN0000020212

Supplementary Figures:

Supplementary Figure 1:

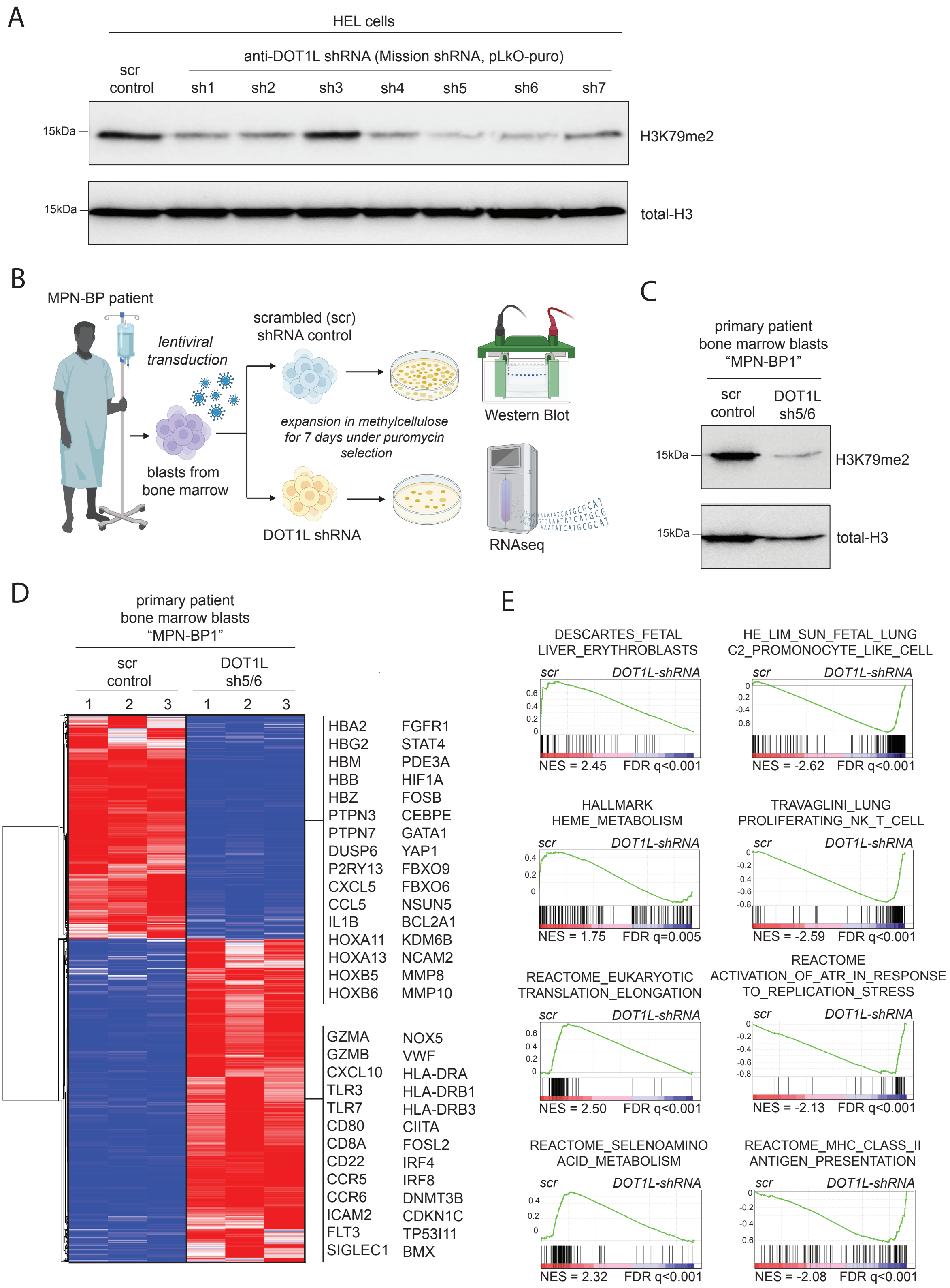

Supplementary Figure 1: (**A**) Western blot showing global H3K79me2 abundance in histone extracts HEL cells transduced with lentiviral particles targeting DOT1L with 7 different shRNAs (Mission shRNA, Sigma/Millipore). ShRNAs 5 and 6 showed the strongest knockdown and were used for further experiments. (**B**) Schematic of experimental workflow to genetically inactivate DOT1L in primary MPN-BP patient cells using RNA-interference. (**C**) Western blot showing global H3K79me2 abundance in histone extracts from primary patient blast cells (MPN-BP1) after 7 days of culture in methylcellulose (H4034-Optimum, Stem Cell Technologies) under selection with puromycin. (**D**) Heatmap of z-scores of differentially expressed genes (padj<0.05; FC>2) in primary patient blast cells (MPN-BP1) after knockdown of DOT1L (shRNA 5/6). (**E**) Selected results from Geneset enrichment analysis (GSEA) of RNAseq data from primary patient blast cells (MPN-BP1).

Supplementary Figure 2:

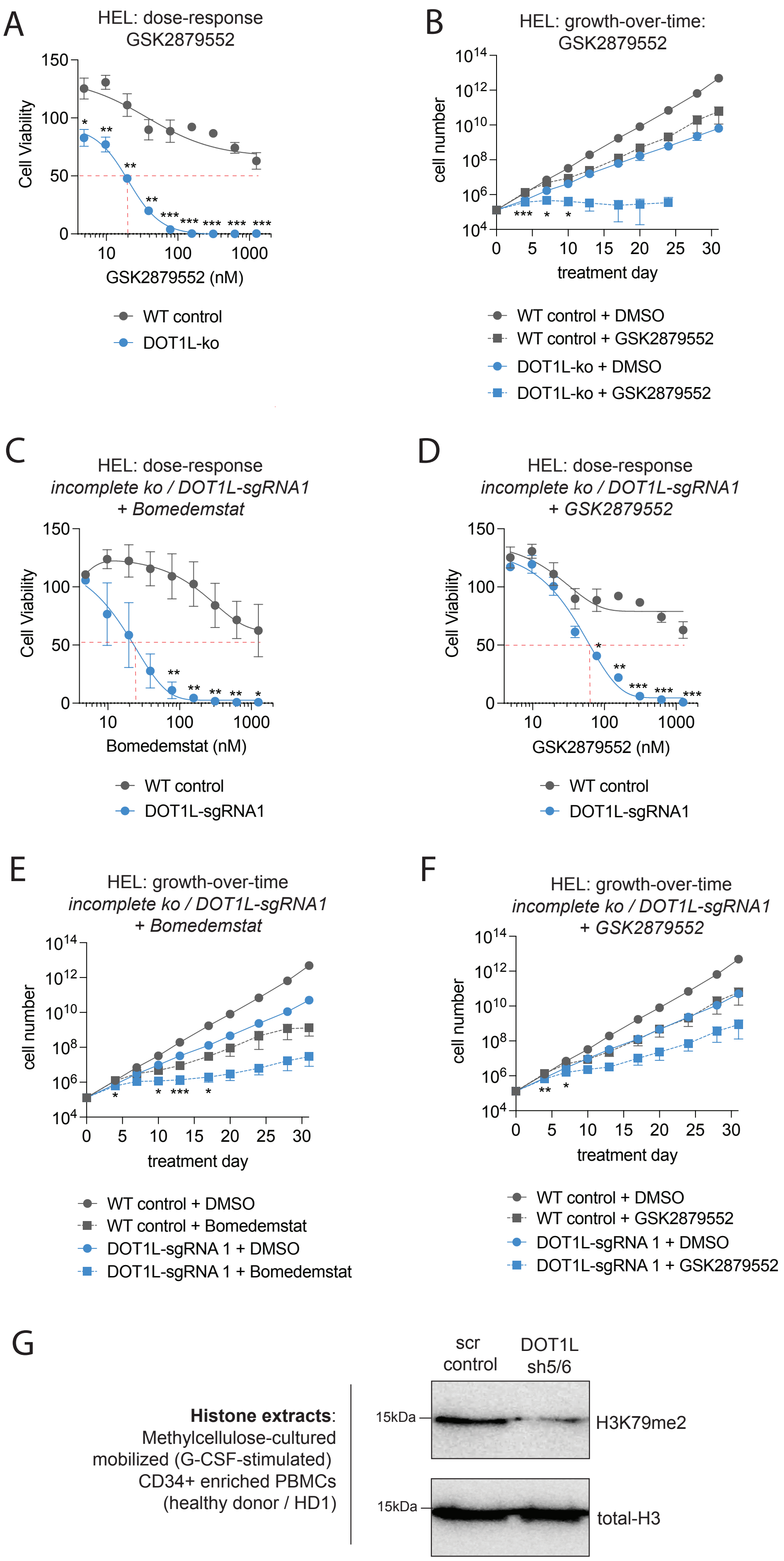

Supplementary Figure 2: (**A**) Dose-response curve showing the sensitivity of HEL cells to GSK2879552 treatment at 6d of incubation determined by MTS assay; n=3 independent experiments, unpaired t-test; *<0.05, **<0.01, ***<0.001. (**B**) Cell growth of HEL cells (empty vector vs. DOT1L-ko, clone 2) in a growth-over-time assay under exposure to GSK2879552 (100nM); unpaired t-test; comparison of DOT1L-ko+Bomedemstat with WT+Bomedemstat; *<0.05, ***<0.001. (**C/D**) Dose-response curves showing the sensitivity of HEL cells (empty vector vs. incomplete knockout / DOT1L-sgRNA1) to (**C**) Bomedemstat) or (**D**) GSK2879552 treatment at 6d of incubation determined by MTS assay; n=3 independent experiments, unpaired t-test; *<0.05, **<0.01, ***<0.001. (**E/F**) Cell growth of HEL cells (empty vector vs. incomplete DOT1L-ko / DOT1L-sgRNA1) in a growth-over-time assay under exposure to (**E**) Bomedemstat (150nM) or (**F**) GSK2879552 (100nM); unpaired t-test; comparison of DOT1L-ko+Bomedemstat with WT+Bomedemstat; *<0.05, **<0.01, ***<0.001. (**G**) Western blot showing global H3K79me2 abundance in histone extracts from normal primary cells from G-CSF mobilized peripheral blood (HD1) after 7 days of culture in methylcellulose (H4034-Optimum, Stem Cell Technologies) under selection with puromycin.

Supplementary Figure 3:

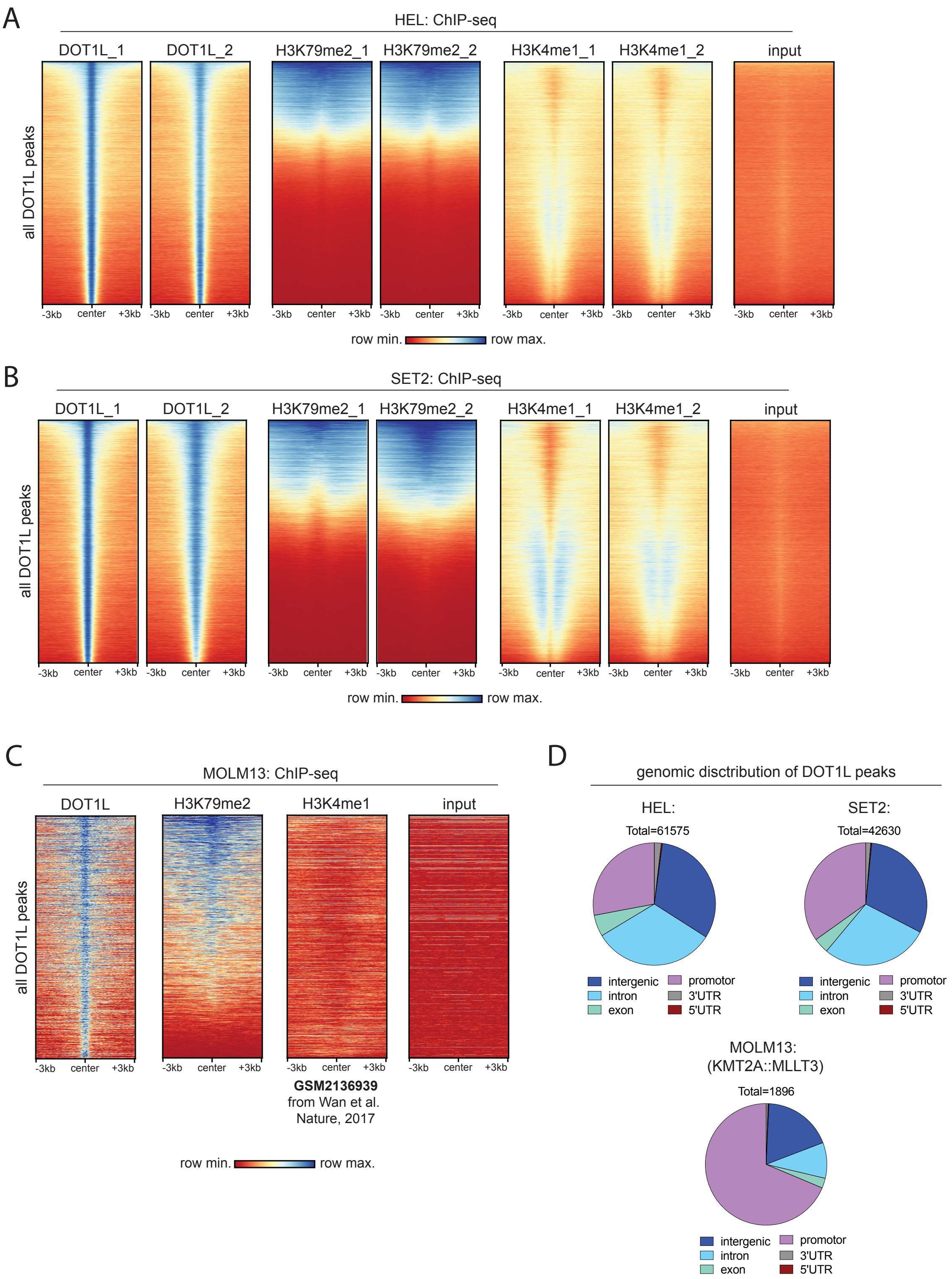

Supplementary Figure 3: (**A**/**B**) Tornado-plots visualizing the chromatin landscape at all DOT1L-bound regions in the genome in (**A**) HEL and (**B**) SET2 cells. Plots show signal intensities of DOT1L, H3K79me2 and H3K4me1 ChIPs in 2 independent replicates around all DOT1L peaks. Maps are sorted by H3K79me2 in a descending order. (**C**) Tornado-plots visualizing the chromatin landscape at all DOT1L-bound regions in the genome in MOLM13 cells. Plots show signal intensities of DOT1L, H3K79me2 and H3K4me1 (H3K4me1 ChIP derived from previously published data / Wan et al., Nature 2017 / GSM2136939). Maps are sorted by H3K79me2 in a descending order. (D) Pie charts showing the genomic distribution of DOT1L peaks in MPN-BP cell lines (HEL and SET2) compared to MOLM13 (KMT2A-rearranged leukemia) cells.

Supplementary Figure 4:

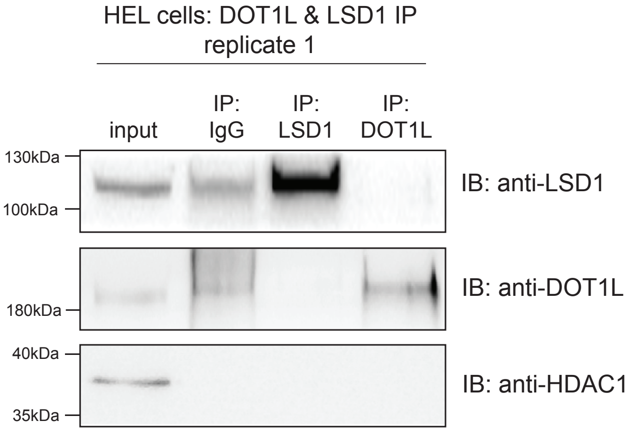

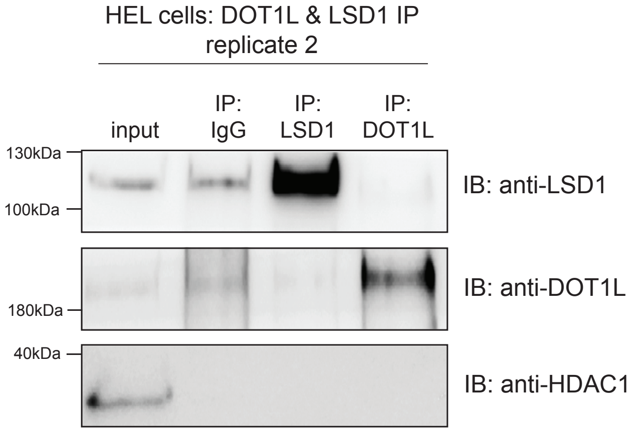

Supplementary Figure 4: Co-Immunoprecipitations of LSD1 and DOT1L were performed from HEL nuclear extracts.

Supplementary Figure 5:

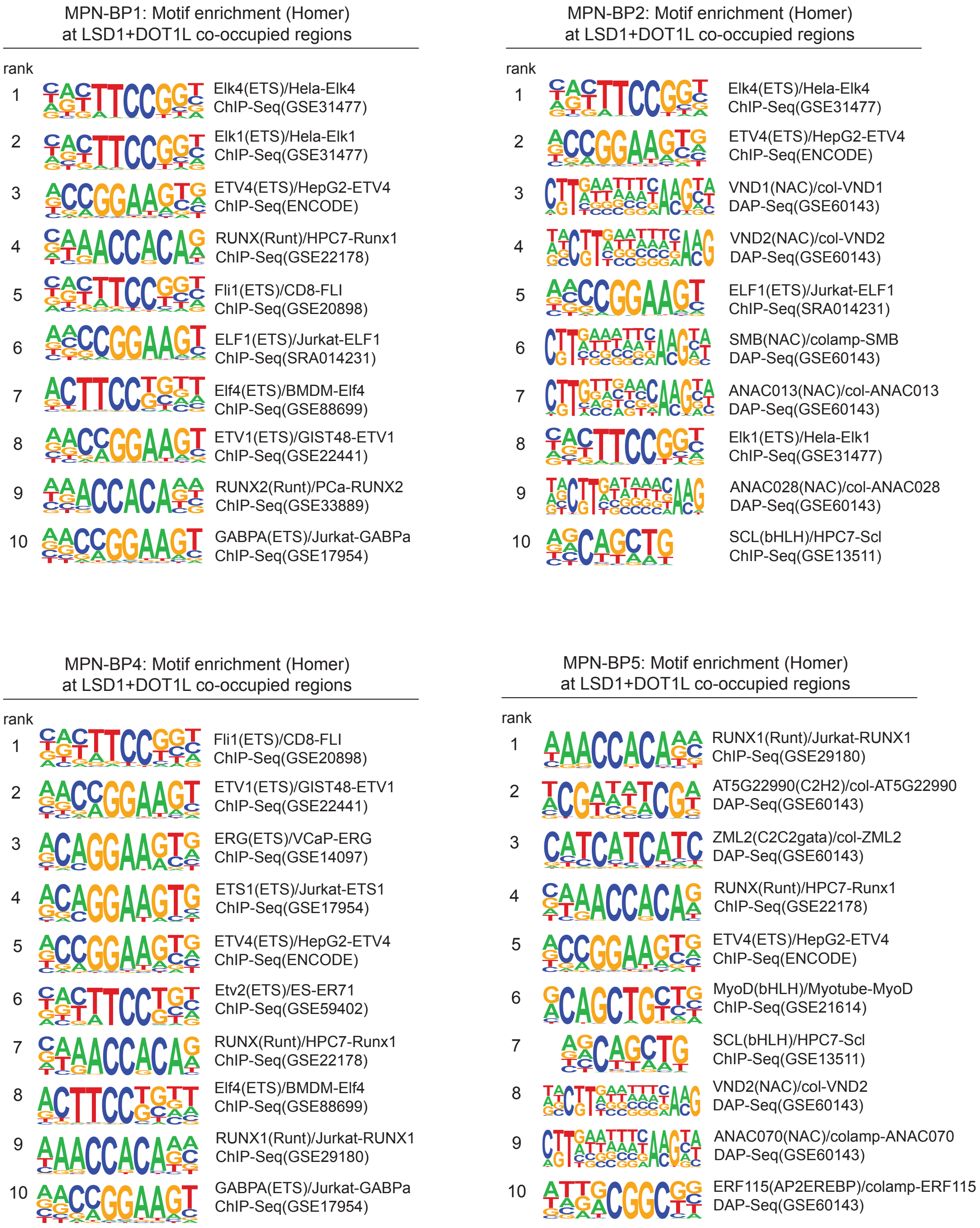

Supplementary Figure 5: Top-10 transcription factor motifs enriched at LSD1 and DOT1L co-occupied regions in 4 MPN-BP primary patient samples. Output of the “Homer” motif enrichment analysis software.

Supplementary Tables:

Supplementary Table 1**:** Complete CRISPR-screening results (MaGECK-Mle output)

Supplementary Table 2: Gene-Ontology Analysis of CRISPR-screening data (STRING-db)

Supplementary Table 3: RNAseq: DESeq2 results (comparison DOT1L-ko vs. WT)

Supplementary Table 4: RNAseq: z-scores and Kmeans clustering of genes related to TOP3000 H3K79me2-bound TSS in HEL cells

Supplementary Table 5: RNAseq: Complete DESeq2 results (comparison DOT1L-shRNA vs. WT in MPN-BP1 primary cells)

Supplementary Table 6: RNAseq: z-scores and Kmeans clustering of DOT1L-shRNA vs. control primary MPN-BP1 cells

Supplementary Table 7: RNAseq: Complete DESeq2 results (comparison Bomedemstat vs. DMSO in DOT1L-ko and WT cells)

Supplementary Table 8: RNAseq: z-scores and Kmeans clustering global expression HEL cells

Supplementary Table 9: DOT1L+LSD1-bound genomic regions (ChIPseeker output) in HEL cells

Supplementary Table 10: DOT1L+LSD1-bound genomic regions (ChIPseeker output): in MPN-BP1 primary cells

Supplementary Table 11: DOT1L+LSD1-bound genomic regions (ChIPseeker output): in MPN-BP2 primary cells

Supplementary Table 12: DOT1L+LSD1-bound genomic regions (ChIPseeker output): in MPN-BP4 primary cells

Supplementary Table 13: DOT1L+LSD1-bound genomic regions (ChIPseeker output): in MPN-BP5 primary cells

Supplementary Table 14: enriched Motifs at DOT1L+LSD1-bound genomic regions (Homer known motifs output) in HEL cells

Supplementary Table 15: enriched Motifs at DOT1L+LSD1-bound genomic regions (Homer known motifs output) in MPN-BP1 cells

Supplementary Table 16: enriched Motifs at DOT1L+LSD1-bound genomic regions (Homer known motifs output) in MPN-BP2 cells

Supplementary Table 17: enriched Motifs at DOT1L+LSD1-bound genomic regions (Homer known motifs output) in MPN-BP4 cells

Supplementary Table 18: enriched Motifs at DOT1L+LSD1-bound genomic regions (Homer known motifs output) in MPN-BP5 cells

Supplementary Table 19: RNAseq: z-scores and Kmeans clustering DOT1L+LSD1 targets
